# Investigation of multifaceted wound healing effect of exopolysaccharide (EPS) produced from probiotic strain *Lactiplantibacillus plantarum* GD2 as in vitro and in ovo

**DOI:** 10.1038/s41598-025-90682-0

**Published:** 2025-10-21

**Authors:** Abdullah Demir, Belma Aslim

**Affiliations:** Department of Biology, Faculty of Science, Gazi University, 06500 Teknikokullar, Ankara, Turkey

**Keywords:** *Lactiplantibacillus plantarum* GD2, Probiotic, Exopolysaccharide, Wound healing, TGF-β1/Smad signaling pathway, Biopolymer, Biotechnology, Cell biology, Drug discovery

## Abstract

Skin wounds may threaten quality of life and cause serious complications. This study aimed to investigate the effects of lyophilized exopolysaccharide (L-EPS) obtained from the probiotic strain *Lactiplantibacillus plantarum* GD2 on various stages of wound healing. The results revealed that L-EPS accelerated in vitro wound healing and increased COL1A1 in L929 cells. L-EPS affected the TGF-β1/Smad signaling pathway by increasing the expression of the TGF-β1, Smad2, Smad3, and Smad4 genes. L-EPS also exerted anti-inflammatory effects by reducing the gene expression of IL-1β, IL-6 and iNOS in TNF-α-induced fibroblasts. Additionally, L-EPS demonstrated fibroproliferative effect on both healthy and TNF-α-induced fibroblasts. Furthermore, L-EPS was found to have a proangiogenic effect in ovo chorioallantoic membrane (CAM) model. This study presents the first-ever characterization of the multifaceted effects of L-EPS derived from the probiotic strain *L. plantarum* GD2 on wound healing. Our findings highlight the potential of L-EPS as effective agent for wound healing and suggest possible application in the development of wound healing biomaterials. By elucidating the mechanism of action of L-EPS in wound healing, this research may provide new perspectives for advanced treatment strategies in the field of wound care.

## Introduction

The skin is the largest organ of the body, consisting of three layers: epidermis, dermis, and hypodermis^1^. The skin separates the body from the external environment and protects the organism from different exogenous threats^2^. Skin disorders, particularly ulcers, are reported to be the third most common reason for clinic admission in developed countries. A wound is described as an injury in the skin, mucous membrane, or tissue arrangement^3^. Wounds are mainly divided into acute and chronic wounds^4^. Acute wounds heal in a predictable time frame, however, chronic wounds have prolonged or non-healing processes^5^. Clinical, economic, and social problems arise as the number of patients increases in tandem with the number of chronic wounds caused by diseases such as diabetes, cancer, and vasculopathy^6^. Current problems and challenges in wound care occur because of : (i) increased costs of wound care, (ii) the increasing number of the aging population, (iii) the presence of comorbidity (e.g., obesity and diabetes) with wound care, (iv) resistance to antimicrobials^7^. Physiological abnormalities within the skin integrity, which is vulnerable to both internal and external factors, can result in limb loss and even death^8^. Systemic antibiotics, various medical dressings, and surgical debridement procedures are commonly utilized in clinical wound care and repair^9^. However, all these interventions have different disadvantages (antibiotic side effects, need for anesthesia, high expenses, etc.). Different studies are ongoing to discover new therapeutic agents and biomaterials that target multiple stages and have fewer side effects in wound healing pathophysiology^6^.

Wound healing is a complicated process that depends on several factors, including the extent of the injury. Repair of acute and chronic wounds requires the organization of cellular and molecular processes, including blood cells, cytokines, and growth factors^10^. Wound healing is characterized by three main phases that overlap in integrated times: inflammation, proliferation, and remodeling phases^11^. When a malfunction occurs in any of these three phases of the healing process, this results in the formation of delayed healing or non-healing chronic wounds^12^.

In the inflammatory phase, proinflammatory mediators are released for control of infection and clear of necrotic tissues, and at the same time, vascular permeability increases^13^. In response to the released chemokines, activated inflammatory cells such as monocytes, lymphocytes, and neutrophils migrate to the wound site^14^.

The proliferation phase of wound healing is characterized by the migration and proliferation of keratinocytes and fibroblasts, angiogenesis, granulation tissue formation, and extracellular matrix (ECM) remodeling^15–18^. At the end of the inflammatory phase and beginning of the proliferative phase, the first fibroblasts appear at the site of injury^19^. Fibroblasts replace the fibrin clot with a diverse ECM, containing components like glycoproteins, proteoglycans, laminin, thrombospondin, glycosaminoglycans, hyaluronic acid, and collagens, playing a pivotal role in guiding fibroblast activity and regulating processes such as angiogenesis and tissue formation^20–22^.

Wounds heal delayed or become chronic when the wound healing process does not continue normally^12^. In general, the healing of chronic wounds is arrested in the inflammatory phase^23^. However, the inflammatory level of wounds, as well as the local levels of growth factors and cytokines, are critical for healing^24^. In the impaired healing process and age-related delayed healing wounds, which are parallel to the changed pro-inflammatory phenotype, high levels of local and systemic tumor necrosis factor-alpha (TNF-α) are detected^25^.

Polysaccharides are components that stimulate wound healing and are included in a variety of wound biomaterials^26^. So far, different wound healing effects of many polysaccharides have been proven (modulation of growth factors and cytokines, pro-angiogenic effect, cell proliferation and migration, re-epithelialization, etc.)^27–31^. Exopolysaccharide (EPS) is a biopolymer composed of sugar monosaccharide residues and sugar derivatives. EPSs secreted into the extracellular environment are classified into two types: homopolysaccharide, which contains a single type of monosaccharide, and heteropolysaccharide, which contains structures such as D-glucose, D-galactose, L-rhamnose, N-acetylglucosamine, N-acetylgalactosamine, and glucuronic acid. EPSs can be produced by plants, fungi, algae, and bacteria^32^.

Studies with EPS obtained from the marine bacterium *Polaribacter*sp. SM1127 was shown that EPS induces cell migration with a strong antioxidant effect in human dermal fibroblasts. Furthermore, this marine EPS promotes full-layer dermal wound closure, which accelerates wound healing^33^. EPS which is produced from marine bacterium *Alteromonas*sp. PRIM-28 was discovered to increase the S phase of the cell cycle and induce the proliferation and migration of keratinocytes and fibroblasts^34^. EPS derived from *Pantoea*sp. YU16-S3 was proven to promote cell adhesion and proliferation on dermal keratinocytes and fibroblasts in cutaneous wound healing. Moreover, this EPS promotes fibroblast migration, accelerates to cell cycle, activates macrophages, increases the expression of heparin-binding EGF, FGF, E-cadherin, and β-catenin, and enhances wound healing via Wnt/β-catenin signaling^35^.

Since lactic acid bacteria (LAB) are safe and beneficial, research with EPSs obtained from them attracts more attention than research with other EPSs^36^. However, only three experimental studies have been reported in the literature about the effects of probiotic-derived EPSs on wound healing. These studies show the antioxidant and antibacterial properties of probiotic EPSs, their effect on in vivo wound closure and collagen deposition, anti-elastase and anti-collagenase activities, and in vitro wound closure^37–39^. Because of their biocompatibility and biodegradability, low cytotoxicity, and chemical functionality, probiotic-derived EPSs have the potential to be active ingredient and wound biomaterial components^40,41^. Despite these potentials, there is no study to date that has revealed the effect of probiotic EPSs on many mechanisms in wound healing phases.

The primary objective of this study is to assess the effects of EPS derived from the probiotic *L. plantarum* GD2 on various phases of wound healing both as in vitro and in ovo. Additionally, our objective is to investigate the potential of *L. plantarum* GD2-derived EPS as both a novel therapeutic agent and a biomaterial component for wound healing.

## Results and discussion

### Effects of L-EPS produced from *L. plantarum* GD2 on L929 cells viability

Fibroblasts are essential cells in all stages of wound healing, and any disturbance in fibroblast activity disrupts wound healing^42^. Therefore, fibroblast survival at the wound site is crucial^43^. In this study, the most effective and non-cytotoxic concentration and treatment time for apply to L929 cells were determined by MTT assay in different conditions (0–1000 µg/mL and 0–36 h). According to results, cell viability was decreased with increasing concentrations of L-EPS and treatment times. However, L-EPS has induced cell death in L929 cells at low rates in the range of 4–20% (*p*< 0.05) (data not shown). According to the report of EL-Adawi et al. (2012), the safe cytotoxicity value of EPS obtained from lactic acid bacteria should be in the range of 10–20%. In this study, we found that even at high concentrations and long time (1000 µg/ml, 36 h), L-EPS maintained cell viability over 80%. EPSs obtained from probiotic lactic acid bacteria are generally regarded as safe (GRAS) according to WHO and the Food and Agriculture Organization (FAO) of the United Nations^44^. In our study, L929 cells were treated with high concentrations of L-EPS and cell viability was not affected highly. These results support the claim that lactic acid bacteria-derived EPS can be used in wound healing. Moreover, the most important features of polysaccharides supporting their usability as biomaterials are that they are biocompatible and biodegradable, as well as having non-toxic effects^45^. Given this information, cell viability results enhance the possibility for non-cytotoxic probiotic EPS to be employed as a biomaterial-improving product in tissue engineering applications^40,41,46^.

### Effect of L-EPS produced from *L. plantarum* GD2 onin vitro wound healing

**Fig. 1:**
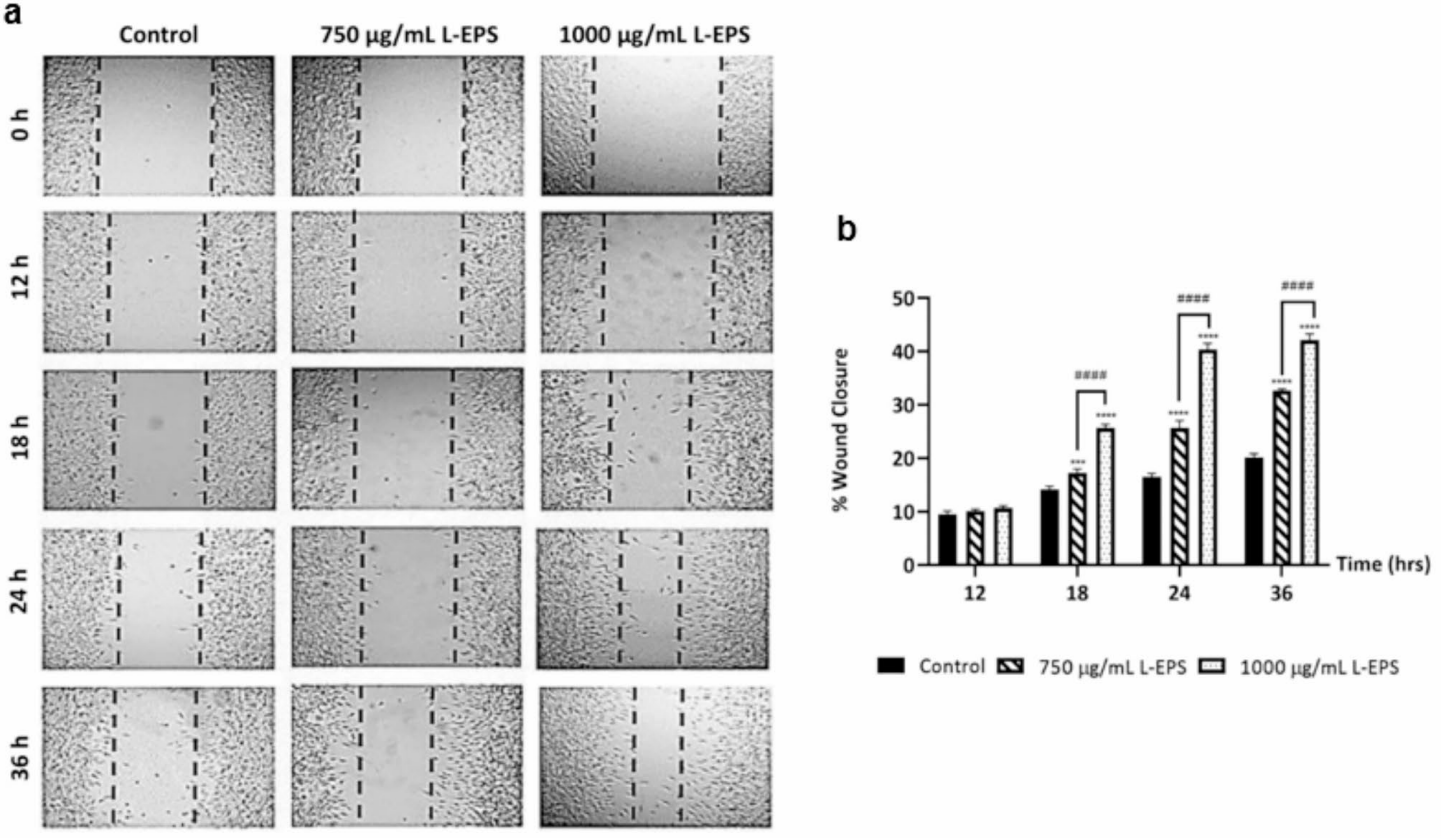
Effect of L-EPS on wound closure in vitro. (**a**) Migration results in the wound model created with L929 fibroblasts. (**b**) Histogram represents the values of wound closure (%). Cells treated with only DMEM were used as the control group. Results, n: 5-well/group, values are expressed as mean ± SD. *p < 0.05, compared with the control group, ^*#*^*p* < 0.05, compared with L-EPS treatment groups.

Since fibroblasts are cell groups that take an active role in the process from the late inflammation phase of the wound repair to the complete healing of the wound, fibroblast migration is a crucial parameter for the initiation of the proliferation phase and a healthy wound healing^47^. An established in vitro scratch assay model was used to determine the effects of L-EPS obtained from the *L. plantarum* GD2 strain on the migration of healthy fibroblast cells L929 at different concentrations (0–1000 µg/mL) and time periods (0–36 h). It was found that L-EPS significantly enhances wound closure with increasing treatment time and concentrations (Fig. 1). The results of in vitro wound analysis also showed that L-EPS has a wound closure-promoting effect ranging from 10.1 to 42.1% in healthy fibroblast cells (*p*< 0.05). In all treatment conditions, higher in vitro wound closure values were detected in the 24 and 36 h treatments of 1000 µg/mL L-EPS compared to the control group. However, the difference in the 24 h and 36 h wound closure results of 1000 L-EPS µg/mL is not remarkable. In wound healing, fibroblasts that migrate to the wound site play an important role in ECM deposition and remodeling^42^, repair of the dermis, and closure of the damaged tissue gap^48^. The effect of probiotic L-EPS to induce fibroblast migration enhances the potentiality of this bioactive polysaccharide to be used in wound healing. There is no previous study in the literature investigating the effect of *L. plantarum* GD2-derived EPS on in vitro wound healing. However, a study with *L. plantarum* EI6-derived EPS found that 24 h and 48 h treatments of probiotic EPS promoted 1.2-fold in human skin fibroblasts compared to the control group^38^. In our study, *L. plantarum* GD2-produced EPS accelerated wound closure by 1.8-fold and 2.5-fold, respectively, compared to the control group after 18 h and 24 h of treatment. Thus, the wound healing potential of GD2-EPS has been detected to be stronger. It can be considered that this bioactive polymer, which is biosafe to use, is an ideal component for pharmacological and therapeutic applications, notably wound healing. Furthermore, the capacity of wound-healing biomaterials to promote cell migration is an essential criteria^49^. The probiotic L-EPS, which is both non-cytotoxic and promotes fibroblast migration, may have the potential to be used as a wound biomaterial-improving polymer as well as a wound healing agent.

### Effect of L-EPS produced from *L. plantarum* GD2 on collagen synthesis

**Fig. 2:**
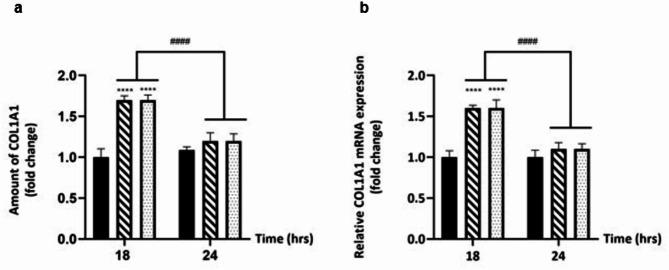
Effect of L-EPS on COL1A1 synthesis (**a**) Fold-changes of COL1A1 amount. (**b**) Fold changes of relative COL1A1 mRNA expression levels. Cells treated with only DMEM were used as the control group. Results, n: 3-well/group, values are expressed as mean ± SD. *p < 0.05, compared with the control group, ^*#*^*p* < 0.05 compared with L-EPS treatment groups.

After the injury to the skin, ECM components are produced to ensure the structural integrity of the wound matrix and to form a temporary connective tissue in the wound area^50^. The ECM is essentially composed of three types of biomolecules: glycosaminoglycans (GAG), proteoglycans, and fibrous proteins (collagen, elastin, fibronectin, etc.)^51^. Collagen, the triple helical protein, is the most abundant in the ECM. Moreover, collagen is vital for cell adhesion and migration, as well as tissue morphogenesis and repair^52^. The ability to synthesize collagen is characteristic of fibroblast^2,53^. In our study, the effect of L-EPS on collagen type 1 alpha 1 (COL1A1) amount was determined by ELISA, while its effect on COL1A1 mRNA expression level was analyzed by qRT-PCR. The results showed that L-EPS increased both the amount and mRNA expression level of COL1A1 (Fig. 2) (*p < 0.05). As a result of L-EPS treatment, the highest increase in COL1A1 amount and gene expression levels was obtained in the 18 h treatment (1.7-fold and 1.6-fold, respectively), and both concentrations were found to have a similar effect. COL1A1 amount and gene expression level results confirmed each other. Although there is no study in the literature showing the inducing effects of EPSs on collagen synthesis, however, there are some studies showing the effects of different polysaccharides on collagen synthesis. Bodin et al.^54^ found that a protein fraction containing 22% polysaccharide derived from the seaweed *Ulva intestinalis* increased collagen synthesis in human dermal fibroblasts ~ 1.5-fold compared to the control grup. In the study by Rioux et al.^55^, galactofucan extracted from brown seaweed *Saccharina longicruris* was found to increase collagen type 1 amount in human fibroblast cells. Esen et al.^56^ reported that low-molecular-weight heparin induces collagen synthesis by fibroblasts in full-thickness surgical incision wounds. Based on the findings, probiotic L-EPS, known for its ability to induce collagen synthesis, may expedite wound closure in acute wounds and facilitate wound healing by increasing decreased extracellular matrix (ECM) synthesis in chronic wounds and promoting tissue regeneration.

Although 18 h L-EPS treatments are prominent in triggering collagen synthesis, according to the results of in vitro wound analysis, the best induction of fibroblast migration was found to be 24 h and 36 h. The migration of fibroblasts to the wound site is strongly impacted by the mechanobiology of the wound matrix. The interaction between migrating fibroblasts and the collagens at the wound site affects fibroblast morphology and ECM modulation^57^. Therefore, triggering collagen synthesis with L-EPS treatment before fibroblast migration to the wound site may have the capability to mechanically prepare the wound bed, allowing fibroblasts to migrate more actively and promoting ECM synthesis to accelerate wound closure. This hypothesis has to be supported by a more advanced molecular and cytological investigation into the mechanobiological regulation of collagen synthesis and fibroblast migration, which is impacted by L-EPS treatment.

### Impact of L-EPS produced from *L. plantarum* GD2 in the TGF-β1/Smad signaling pathway

**Fig. 3:**
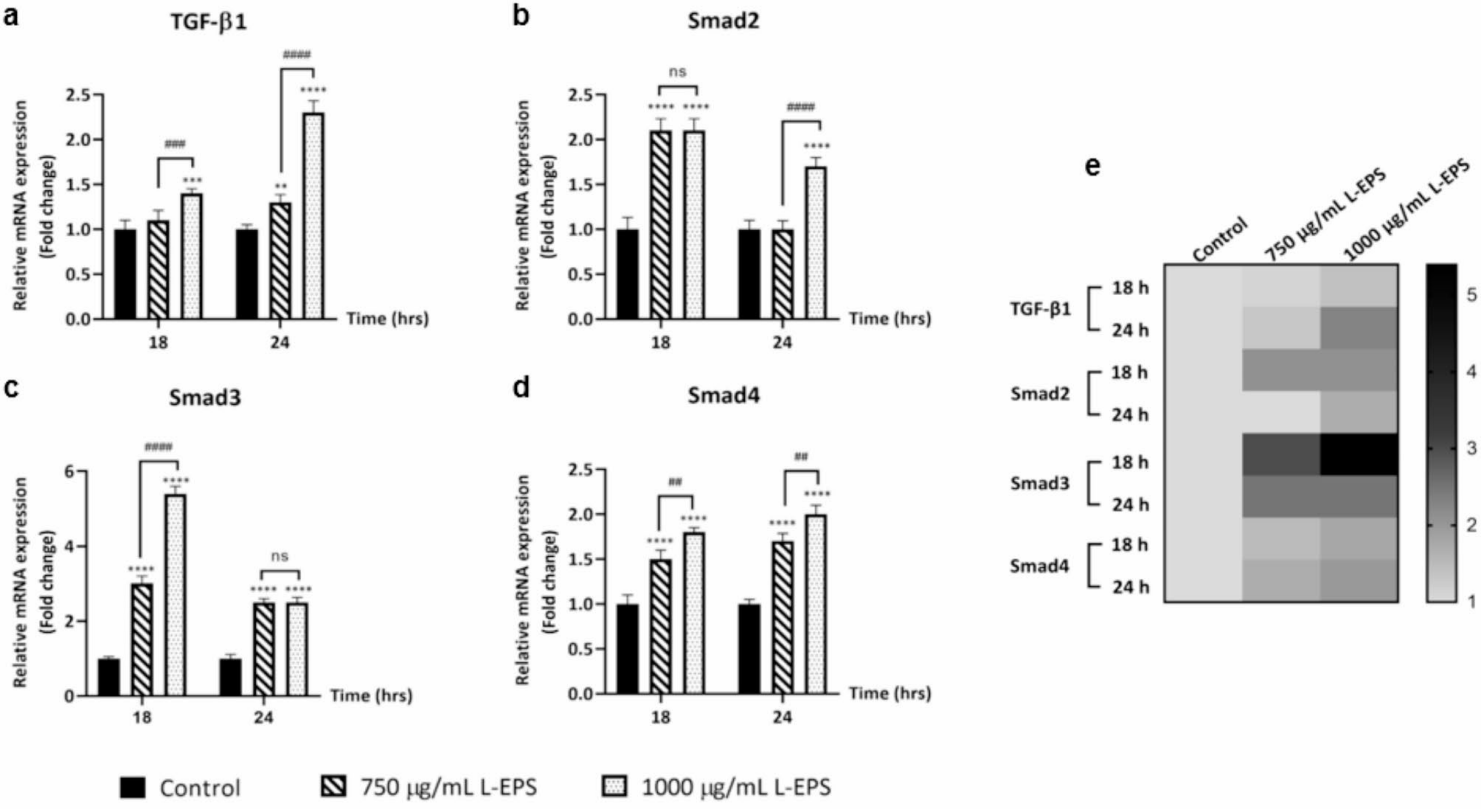
Effect of L-EPS on the TGF-β1/Smad signaling pathway. The relative mRNA expression levels of TGF-β1 (**a**), Smad2 (**b**), Smad3 (**c**), and Smad4 (**d**) in L929 cells. (**e**) Heat map of TGF-β1/Smad pathway-related genes. Cells treated with only DMEM were used as the control group. Results, n: 3-well/group, values are expressed as mean ± SD. *p < 0.05, compared with the control group, ^*#*^*p* < 0.05, compared with L-EPS treatment groups.

TGF-β1 is a cytokine that has key pathophysiological functions in mammals. It is a multifunctional signaling molecule that controls cell proliferation, differentiation, morphogenesis, tissue homeostasis, and regeneration^58,59^. Furthermore, TGF-β1/Smad pathway is an essential signaling pathway for wound healing, with many effects such as cell proliferation and migration, inflammation, and ECM regulation^60^. For these reasons, it is critical to determine whether L-EPS treatment modulates this signaling via fibroblast cells to demonstrate that this biopolymer is a useful agent in wound healing. Therefore, to clarify the relationship of L-EPS with this pathway, RT-PCR analysis of TGF-β1, Smad2, Smad3, and Smad4 genes was performed in L929 cells. Results were shown that 18 h of treatment of 750 µg/mL L-EPS had no significant effect on TGF-β1 mRNA expression, while 24 h of treatment had no significant effect on Smad2 mRNA expression (*p* > 0.05). However, treatment with 1000 µg/mL L-EPS (18 h and 24 h) significantly increased the expression levels of TGF-β1 (1.4–2.3-fold), Smad2 (2.1–1.7-fold), Smad3 (5.4–2.5-fold) and Smad4 (1.8–2.0-fold) in L929 fibroblasts (Fig. 3) (*p < 0.05). Our results showed that 1000 µg/mL L-EPS was more effective in TGF-β1/Smad signaling pathway compared to 750 µg/mL L-EPS treatment. Despite the fact that no research has been reported on the effects of EPSs on the TGF/Smad pathway, there have been a few studies on the effects of different polysaccharides on this pathway. Zhang et al.^61^ reported that polysaccharide-based hydrogel containing mannitol and glucose residues from *Bletilla striata* increased TGF-β1, Smad2, and Smad3 mRNA expression levels, induced collagen production via TGF-β1/Smad signaling pathway, and accelerated wound healing. You et al.^62^ found that heteroglycan polysaccharides obtained from *Panax notoginseng* root induced procollagen synthesis with increasing TGF-β1, Smad2, Smad3, and Smad4 mRNA expression levels in human dermal fibroblasts in which oxidative damage was created by hydrogen peroxide. As emphasized in the literature, it was noted that the triggering of the TGF-β1/Smad signaling pathway and the increase in COL1A1 synthesis and wound closure were paralleled with L-EPS treatment in our study. Accordingly, it can be said that probiotic-derived EPS provides migration and wound closure by playing a role in COL1A1 synthesis via inducing TGF-β1/Smad signaling pathway.

In our results, Smad3 was found to be the gene most affected by L-EPS in the TGF-β1/Smad pathway. Smad3 modulates wound inflammation by suppressing the expression of inflammation-induced increased peroxisome proliferator-activated receptor (PPAR) and thereby controlling the timely suppression of wound inflammation^63^. Based on our study, it is thought that L-EPS derived from *L. plantarum* GD2 may shows a modulatory effect on wound inflammation via Smad3. Moreover, Smad3 was found to be higher expressed with L-EPS treatment at 18 h. Depending on L-EPS treatment, the early response of Smad3 may have the potential to modulate inflammation, which is the first stage of wound healing, and also heal chronic wounds trapped in the inflammation stage. This view needs to be supported by showing in detail how the relationship between Smad3 and the inflammation phase is affected with L-EPS treatment.

### Anti-inflammatory effects of L-EPS produced from *L. plantarum* GD2

**Fig. 4:**
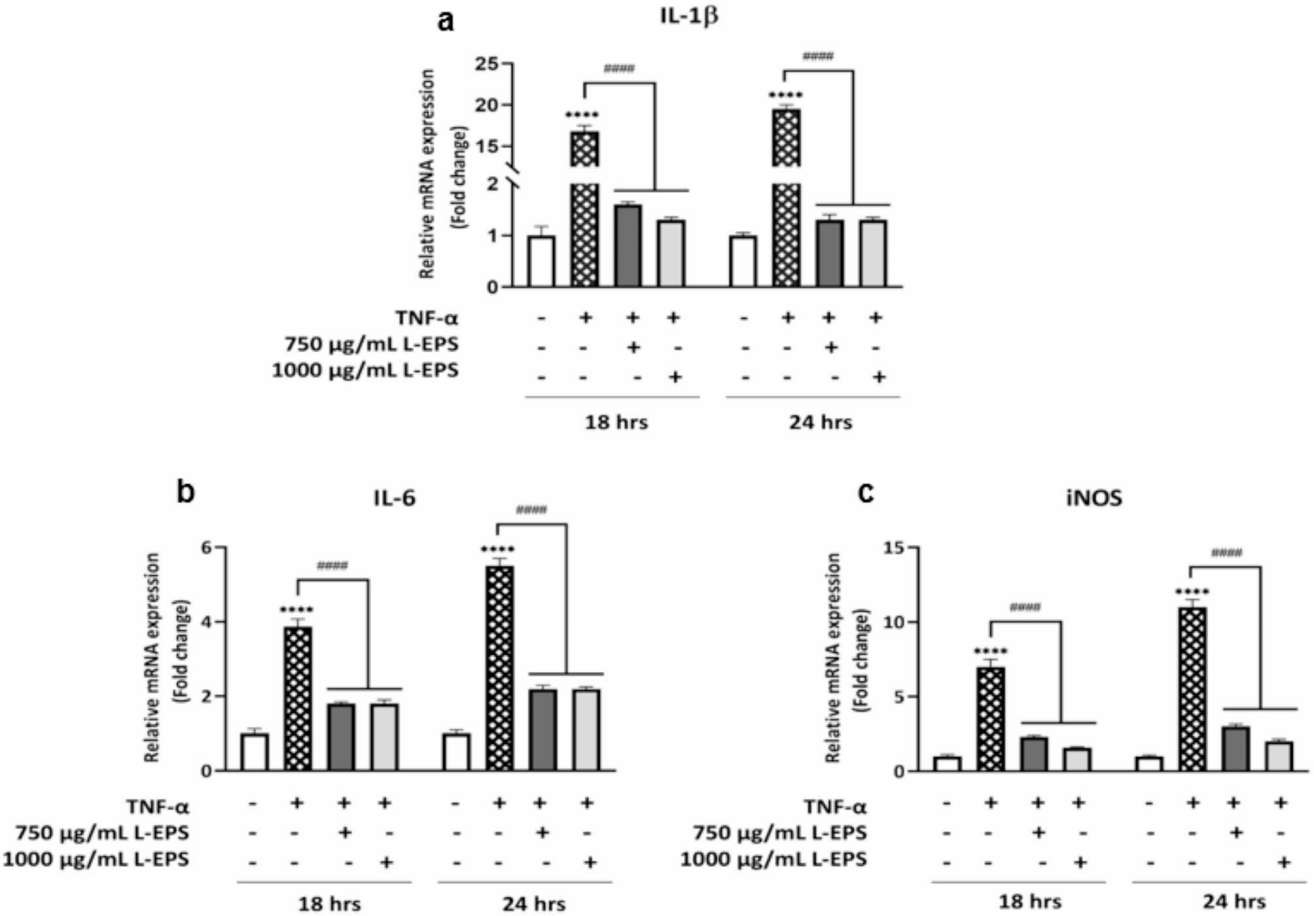
Effects of L-EPS on IL-1β (**a**), IL-6 (**b**), and iNOS (**c**) mRNA expressions in TNF-α-induced L929 cells. Cells treated with only DMEM were used as the control group. Results, n: 3-well/group, values are expressed as mean ± SD. *p < 0.05, compared with the control group, ^*#*^*p* < 0.05, compared with the TNF-α-treated group.

Wound healing is delayed when it does not proceed normally, as in acute wounds, and wounds become chronic. The inflammatory phase continues in chronic wounds, and wound healing interruptions during the regeneration^64^. The high concentration of the pro-inflammatory cytokine tumor necrosis factor-alpha (TNF-α) in wounds is one of the factors causing these disruptions in skin wound healing. In our study, the effects of EPS obtained from probiotic on IL-1β and IL-6, and iNOS genes were investigated by qRT-PCR in an in vitro inflammation model created in L929 fibroblast cells with TNF-α. At the beginning of the study, the non-toxic and effective TNF-α concentration was determined by the MTT method. According to our results, the appropriate non-toxic concentration to be used in the study was found to be 20 ng/mL (data not shown). As shown in Fig. 4, the treatment of L929 cells with TNF-α significantly increased the expression of IL-1β, IL-6, and iNOS mRNA compared with the control group (*p < 0.05), while L-EPS treatments were markedly decreased mRNA expression of these genes with compared with the TNF-α group (^*#*^*p*< 0.05). High TNF-α concentrations are associated with impaired healing processes, particularly in diabetic wounds, and wound healing delay is characterized by TNF-α-induced fibroblast apoptosis and fibroblast proliferation inhibition^65–67^. In addition, high TNF-α concentration inhibits the production of ECM proteins such as collagen type 1 and fibronectin. This causes a decrease in ECM deposition and a delay in wound healing^68^. The decrease of pro-inflammatory cytokines (IL-1β, IL6) expression with L-EPS treatment indicates that this bioactive polymer possesses anti-inflammatory properties. Increased nitric oxide (NO) production in chronic wounds such as venous leg ulcers causes delayed wound healing through peroxynitrite signaling and damage to granulation tissue^69,70^. Inducible nitric oxide synthase (iNOS), which is responsible for NO production, is synergistically stimulated by pro-inflammatory cytokines such as TNF-α, IL-1, and IL-6. Besides, high NO concentration in chronic wounds causes decreased collagen synthesis^68^. Because of these reasons, iNOS overexpression is another major factor in chronic wounds and delayed healing^71^. As a result, probiotic-derived EPS may reduce NO production in chronic wounds with decreasing iNOS mRNA expression, increase collagen synthesis, and improve in the healing of chronic wounds. In chronic wounds with TNF-α/nuclear factor-kappa B (NF-B) activity, it is possible to reduce inflammation and accelerate wound healing via the TGF-β1/Smad signaling pathway^72^. In light of this information, probiotic-derived EPS can accelerate wound healing by suppressing inflammation in chronic wounds through the TGF-β1/Smad pathway. Taken together, in addition to the use of L-EPS as an anti-inflammatory wound-healing agent, there may also be potential for this probiotic-derived bioactive polymer to be used as a wound-healing anti-inflammatory biomaterial product. Moreover, according to our study, L-EPS mostly increased Smad3 mRNA expression in the TGF-β1/Smad pathway (Fig. 3). Smad3 modulates wound inflammation by suppressing the expression of peroxisome proliferator-activated receptor (PPAR), which increases during inflammation, and thus provides control of wound inflammation^63^. According to this report, L-EPS may have an anti-inflammatory effect via Smad3.

### Fibroproliferative effects of L-EPS produced from *L. plantarum* GD2

**Fig. 5:**
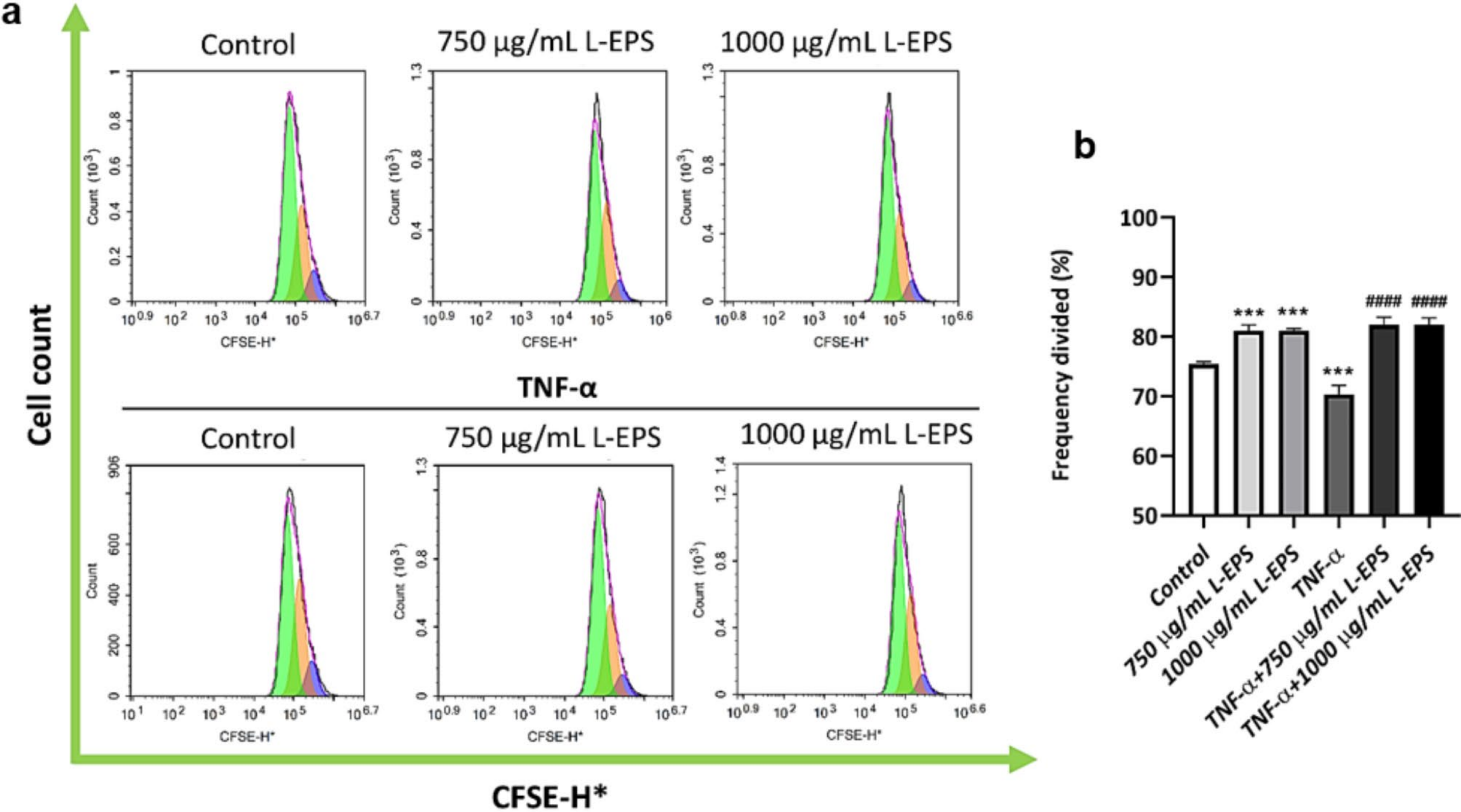
Proliferative effect of L-EPS on both healthy and TNF-α-induced L929 fibroblasts. (**a**) Flow cytometry proliferation results. (**b**) Histogram represents the values of frequency divided (%). Cells treated with only DMEM were used as the control group. Results, n: 3-tube/group, values are expressed as mean ± SD. *p < 0.05, compared with the control group, ^*#*^*p* < 0.05, compared with the TNF-α-treated group.

Fibroblast proliferation is critical for filling the tissue space formed after injury with proliferating fibroblasts and synthesizing the ECM, which is destroyed by an increase in the number of fibroblasts in the wound area^73–76^. TNF-α levels in circulating plasma are generally around 11 pg/mL while in healthy skin tissue TNF-α levels are about 1.5 ng/mL^77,78^. Furthermore, elevated TNF-α levels have been found in chronic wound fluids^79^. Furthermore, high TNF-α concentrations cause dermal fibroblast apoptosis, which impairs wound healing^69^. In our study, the proliferative effects of L-EPS treated on both healthy and TNF-α-induced L929 cells were determined by flow cytometry. The results showed that the control group cells were divided 75.44% in 24 h. The cells treated with L-EPS showed an equivalent proliferative effect at both doses, and 81% of the cells were divided (*p < 0.05). In contrast, 70% of L929 fibroblasts treated with 20 ng/mL TNF-α were divided, and proliferation was determined to be reduced when compared to the control group (*p < 0.05). L-EPS treatment to TNF-α-induced L929 cells was found to have an equal proliferative effect at both concentrations (82%) compared to the TNF-α treated group (Fig. 5) (^*#*^*p* < 0.05). These results indicate that the probiotic EPS has a proliferative effect in both healthy and TNF-α-induced L929 fibroblasts. According to our results, probiotic derived-EPS might be a natural therapeutic agent that can be used in wound healing by inducing fibroblast proliferation. The fact that L-EPS stimulates proliferation in addition to inducing collagen synthesis and fibroblast migration supports our hypothesis that this biopolymer can enhance wound healing via the TGF-β1/Smad signaling pathway. Moreover, probiotic-derived EPS may have the potential to be used as proliferative agents in chronic wounds with high tissue loss as a result of decreased fibroblast proliferation due to high TNF-α concentration and triggering of fibroblast apoptosis, such as diabetic wounds. In order to support this view, the histological, cytological, and molecular effects of L-EPS in different chronic wound models need to be investigated.

### Pro-angiogenic effects of L-EPS produced from *L. plantarum* GD2

**Fig. 6:**
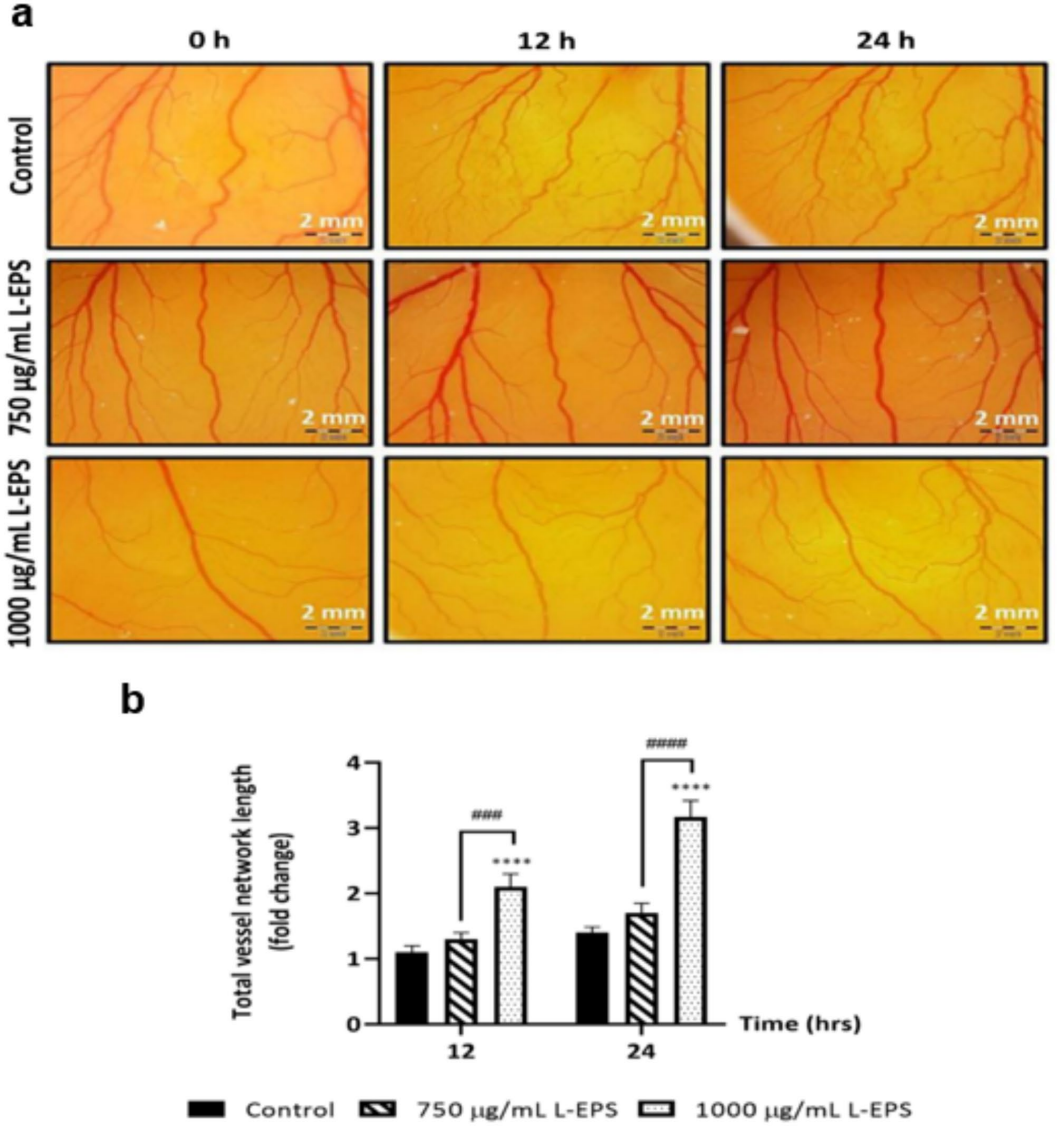
Pro-angiogenic effect of L-EPS. (**a**) Microscopic visualization of the pro-angiogenic effect in the CAM model (1x). (**b**) Total vessel network length levels. Fertilized chicken eggs treated with only 1xPBS were used as the control group. Results, n: 3-egg/group, values are expressed as mean ± SD. *p < 0.05, compared with the control group, ^*#*^*p* < 0.05 compared with L-EPS treatment groups.

Angiogenesis is crucial for wound healing. Blood vessels generated in the damaged tissue transport nutrients and oxygen to the injured tissue^80^. The creation of new capillaries and the repair of damaged blood vessels are followed by the establishment of a new vascular network in the injured tissue following the injury^81^. One of the major difficulties underlying the pathophysiology of chronic wounds is insufficient perfusion in tissue damage after injury. Although the application of pro-angiogenic growth factors and vascularized scaffolds aids neovascularization, innovative therapeutic techniques that enable revascularization relevant to the wound microenvironment are still being researched^82,83^. In our study, the pro-angiogenic effect of L-EPS was investigated using the in ovo chorioallantoic membrane (CAM) model. CAM assay results are shown in Fig. 6. Results showed that 750 µg/mL L-EPS treatment for 12 h and 24 h increased the total vessel length folds by 1.3 (*p* > 0.05) and 1.7 fold (*p < 0.05), respectively. However, it was determined that 1000 µg/mL L-EPS treatment for 12 h and 24 h increased the total vessel length folds by 2.1 and 3.2 fold, respectively (*p < 0.05). The highest pro-angiogenic effect of L-EPS was detected at 1000 µg/mL for 24 h. There is no study in the literature that shows the pro-angiogenic effects of probiotic-derived applications and different polysaccharides in the CAM model, however, there are studies conducted in other models. Matou et al.^84^ studied the pro-angiogenic effects of oversulfated EPS in heteropolysaccharide structure derived from the mesophilic bacterium *Alteromonas infernus*. Oversulfated mesophilic bacterial EPS has been found to increase the levels of fibroblast growth factor-2 (FGF-2) and vascular endothelial growth factor (VEGF), which are responsible for inducing angiogenesis, in an in vitro tubular model created with human umbilical vein endothelial cells (HUVECs), therefore, mesophilic bacterial EPS may have a pro-angiogenic effect. Marinval et al.^85^ showed that the oversulfated fraction of fucoidan derived from the seaweed *Ascophyllum nodosum* has pro-angiogenic effect by stimulating vascularization in HUVECs. Jiang et al.^86^ reported that a sulfated porphyry-like polysaccharide derived from the red seaweed *Bangia fuscopurpurea*significantly inhibited the VEGF receptor kinase inhibitor in HUVEC cells and had a pro-angiogenic effect. One of the most important reasons underlying the pathophysiology of chronic wounds such as diabetic, venous, and ischemic ulcers is chronic hypoxia and micronutrient conduction disorder due to impaired angiogenesis^17^. Due to the inadequacy of the current angiogenesis-targeted therapeutic agents used in chronic wounds, the investigation of active substances that may have pro-angiogenic effects is still ongoing^87,88^. L-EPS, which has a pro-angiogenic effect, can support angiogenesis mechanisms in chronic wounds and provide chronic wound closure by carrying oxygen and micro-nutrients to the wound tissue via the formation of new blood vessels. Furthermore, L-EPS may have a faster wound closure potential than its equivalents by promoting revascularization in the wound region in acute wounds.

The fundamental features sought in biomaterials that have the potential to be used in wound healing are biocompatibility, biosafety, biodegradability, non-toxicity, promoting cell migration and proliferation, anti-inflammatory properties, rough surface area to promote cell motility, and adhesion, microporosity to enable cell invasion and endothelial sprouting^49,89–92^. This study showed that *L. plantarum* GD2-derived L-EPS is non-toxic, stimulates migration and proliferation, and has an anti-inflammatory effect on genes associated with wound inflammation. In addition, our team investigated the nano-scale topological properties of L-EPS obtained from *L. plantarum*GD2 strain in a different study. According to the findings of related study, L-EPS features a rough surface area in atomic force microscope (AFM) photomicrographs and a porous structure in scanning electron microscope (SEM) photomicrographs^93^. In the light of this information, it is considered that the biocompatible and biosafe L-EPS has a high potential to be a biomaterial that can be used in wound healing. However, further research into the physico-chemical characteristics of L-EPS in relation to the requirements for becoming a biomaterial is required to support this viewpoint.

## Conclusion

In conclusion, our study demonstrated that L-EPS originated from the *L. plantarum* GD2 strain exhibits in vitro wound healing and in ovo pro-angiogenic effects. Importantly, it was found that probiotic L-EPS does not induce cytotoxicity in L929 cells, even at high concentrations and treatment durations. Based on the results, it is proposed that the biosafe L-EPS promotes fibroblast-mediated wound healing by facilitating fibroblast migration and collagen synthesis through the TGF-β1/Smad signaling pathway. Furthermore, its observed that L-EPS suppresses genes associated with wound inflammation in TNF-α-mediated inflammation. Our results, it has demonstrated the proliferative effect of L-EPS on fibroblast cells, both under healthy and inflammation-triggered conditions. Additionally, it was demonstrated that probiotic-derived L-EPS may have a pro-angiogenic effect. These results provide the first-ever evidence in the literature that *L. plantarum*-derived EPS promotes wound healing through the TGF-β1/Smad signaling pathway.

Biocompatibility, biosafety, biodegradability, non-toxicity, stimulation of cell migration and proliferation, and anti-inflammatory activities are among the key criteria sought in wound healing biomaterials. Based on the comprehensive results of our study, it can be predicted that the probiotic-originated L-EPS used not only possesses potential as an active ingredient for wound healing but also can hold promise as a biomaterial product in wound healing applications. Furthermore, it has beneficial properties in promoting wound healing make it a compelling candidate for further exploration in wound healing therapies.

## Materials and methods

### Bacterial strain and culture conditions

The *Lactiplantibacillus plantarum*GD2 strain, identified by 16 S rRNA and biochemical analyses, was used in this study. This strain isolated from healthy infant feces was obtained from the Gazi University, Biotechnology Laboratory Collection for Type culture collection. The definitions of morphological, cultural, and biochemical characteristics of this strain were shown in previous studies of our team^93–95^. In previous studies, the *L. plantarum* GD2 strain was selected as a high EPS producer. Characteristic and structural properties of EPS derived from *L. plantarum*GD2 was revealed^93–95^. Working cultures were prepared from frozen stocks by twice activated in MRS broth. Incubations of lactobacilli were conducted at 37 °C for 18 h. *Lactobacilli*with an optical density of 0.6 at 600 nm (~ 8.5 log CFU/ml) were used in the studies. The strain was stored in MRS broth with 10% glycerol (Sigma-Aldrich) at − 40 °C to use in other studies^96^.

### EPS isolation, lyophilization, and quantification

Cultures were heated at 100 °C for 15 min. Cold bacterial cultures were treated with a 17% (v/v) 85% trichloroacetic acid (TCA) (Merk Millipore) solution before being centrifuged for 20 min at 13.000 rpm. This step enabled the removal of cells and proteins from the medium. EPSs were centrifuged for 15 min at 13.000 rpm after overnight incubation with 1 volume of cold pure ethanol (Sigma-Aldrich). The precipitation with ethanol was repeated. The EPS-containing pellet was then suspended in deionized water and lyophilized (Christ alpha 2–4 LD Plus freeze Dryer)^97^. The total carbohydrate amount of lyophilized-EPSs (L-EPSs) was calculated using the phenol-sulfuric acid method. For EPS quantification, glucose (Merck Millipore) was used as a standard^98^.

### Cell culture conditions and treatment

L929 mouse fibroblasts (ATCC CCL-1, NCTC Clone 929) were cultured in Dulbecco’s Modified Eagle Medium (DMEM) (Capricorn Scientific) supplemented with 10% heat-inactivated fetal bovine serum (FBS) (Gibco), penicillin/streptomycin (100 U/mL: 100 µg/mL) (Gibco). Cells were cultured in the prepared culture medium and 37 °C 5% CO_2_incubator^99^. The culture media was replaced every 2–3 days after the cells were cultured. Cells reaching 80–90% density were collected with 0.25% trypsin/EDTA (Capricorn Scientific) and used in studies. Lyophilized EPS (L-EPS) were dissolved in serum-free DMEM and filtered with a sterile filter with a pore diameter of 0.22 μm (Sigma-Aldrich). In this study, cells were treated with L-EPS concentrations of 750 and 1000 µg/ml for 0–36 h. Recombinant mouse TNF-α (Abcam) was used to create an in vitro fibroblast inflammation model. The model was constructed as follows: L929 cells were seeded into 12- or 6-well plates (MilliporeSigma), which were then allowed to equilibrate overnight before experiments. Recombinant mouse TNF-α was prepared at 20 ng/mL and treated to cells for 30 min. After incubation, the medium containing TNF-α is removed^100^. Then, cells were treated with L-EPS as described above.

### Determination of cell viability

After treatment with the indicated L-EPS and/or TNF-α, all the culture media in a 96-well plate (MilliporeSigma) were removed and replaced with FBS-free 0.5 mg/mL of MTT (ThermoFisher Scientific) and L929 cells were incubated for 4 h at 37 °C in a CO_2_ incubator. The MTT solution was removed and 200 µL of dimethyl sulfoxide (DMSO) (Sigma-Aldrich) was added to all wells. Cells incubated in a CO_2_incubator for 30 min at 37 °C were read at 570 nm in a microplate reader (Biotek Instruments). The viability of the untreated control group cells was 100%. The cell viability of the treatment groups was determined by comparing the absorbance values with the control group^101^.

### In vitro wound healing assay

The study of Liang et al. was followed for the scratch assay protocol^102^. 5 × 10^5^cells/well into a 24-well plate (MilliporeSigma) were seeded. After an incubation period of 18–24 h, an artificial wound site was created in each well with p200. After wound formation, the medium containing the cell debris was washed with serum-free DMEM medium. Then, the cells were treated for 0–36 h with 750 and 1000 µg/mL L-EPS. DMEM medium was only applied to control groups at the indicated times. The migration of fibroblasts to the wound site was analyzed by area measurement with ImageJ Software (U. S. National Institutes of Health, USA). The percentages of wound closure were calculated according to the following formula^103^:

$${\text{migration}}\;{\text{rate}}\left( {\text{\% }} \right) = \frac{{{\text{distance}}\;{\text{within}}\;{\text{scratch}}\left( {0\;{\text{h}}} \right) - {\text{distance}}\;{\text{within}}\;{\text{scratch}}\left( {{\text{t}}.\;{\text{h}}} \right)}}{{{\text{distance}}\;{\text{within}}\;{\text{scratch}}\left( {0\;{\text{h}}} \right)}} \times 100$$

### Enzyme-linked immunosorbent assay (ELISA)

The concentration of collagen type 1 alpha 1 (COL1A1) released by the L929 cells were measured using a mouse COL1A1 ELISA kit (Cloud-Clone Corp.) according to the manufacturer’s instructions. The seeded cells were treated with 750 and 1000 µg/mL L-EPS for 18 and 24 h. After incubation, samples were collected and the protocol was followed. Samples were read in a microplate (Biotek Instruments) reader at 450 nm. Results were calculated according to the standard graph^104^.

### Reverse transcription-polymerase chain reaction (RT-PCR)

After cell treatments, total RNA isolation from L929 fibroblast was performed using GeneJET RNA purification kit (Thermo Fisher Scientific) according to the manufacturer’s instruction. Absorbance values of 260 and 280 were measured in order to calculate the purity and quantification of the isolated total RNA samples. cDNA was synthesized from 1 µg of total RNA using the High-Capacity cDNA Reverse Transcription Kit (Thermo Fisher Scientific). After cDNA synthesis, the relative expression of the targeted genes in the study was determined using Applied Biosystems® QuantStudio® 3 Real-Time PCR System (Thermo Fisher Scientific) thermal cycler. SensiFAST™ SYBR® Lo-ROX Kit (Bioline) protocol was performed for qRT-PCR analysis. PCR was carried out at 95 °C for 3 min, 95 °C for 30 s, and 60–63 °C for 30 s for 40 cycles. All amplification reactions for each sample were performed in triplicate and repeated at least 3 times. The glyceraldehyde 3-phosphate dehydrogenase (GAPDH) gene was used as the endogenous control. After standardization to GAPDH, the relative mRNA expressions were quantified using the 2^−ΔΔCt^ technique^105^. The primer sequences for the genes used in the study are presented in Table 1.

**Table 1:** Primers used in RT-PCR.

Genes (Mus musculus)	Primers (5’→3’)	Length (bp)	Annealing temperature (°C)
COL1A1 ^106^	FW: ACATGCCGCGACCTCAAGAT RV: ATGTCTAGTCCGAATTCCTG	20 21	60
TGF-β1 ^107^	FW: GTGTGGAGCAACATGTGGAACTCTA RV: TTGGTTCAGCCACTGCCGTA	25 20	60
Smad2 ^107^	FW: AACCCGAATGTGCACCATAAGAA RV: GCGAGTCTTTGATGGGTTTACGA	23 23	60
Smad3 ^107^	FW: GTCAACAAGTGGTGGCGTGTG RV: GCAGCAAAGGCTTCTGGGATAA	21 22	60
Smad4 ^107^	FW: TGACGCCCTAACCATTTCCAG RV: CTGCTAAGAGCAAGGCAGCAAA	21 22	60
IL-1β ^108^	FW: GCAACTGTTCCTGAACTC RV: CTCGGAGCCTGTAGTGCA	18 18	62.5
IL-6 ^109^	FW: GTACTCCAGAAGACCAGAGG RV: TGCTGGTGACAACCACGGCC	20 20	63
iNOS ^110^	FW: TAGGCAGAGATTGGAGGCCTTG RV: GGGTTGTTGCTGAACTTCCAGTC	22 23	63
GAPDH ^111^	FW: GACCCCTTCATTGACCTCAA RV: CTTCTCCATGGTGGTGAAGA	20 20	60

### Detection of fibroblast proliferation using flow cytometry

Proliferation analysis was performed using the CellTrace™ Cell proliferation kit (Thermo Fisher Scientific). First, untreated cells were harvested and treated with 10 µM CSF proliferation dye. Cells were then seeded at ~ 2 × 10^5^/well in a 6-well plate. Before treatments, cells were washed with phosphate-buffered saline (PBS) and treatments were performed. Cell proliferation was detected in Flow Cytometry (NovoCyte) according to the amount of dye in the nuclei of the cells^112^. Only 24 h of L-EPS treatment was analyzed so that L929 cells were not analyzed below the doubling time.

### In ovo chick chorioallantoic membrane (CAM) assay

The in ovo CAM model was used to evaluate the pro-angiogenic effect of L-EPS. In the study, fertilized chicken eggs were provided from Bil-Yem Food Ind. Trade. Co. Ltd. After the eggs were incubated at 37 ^o^C for 3 days at 70% humidity in a hatcher (Europe), ~ 10 mL of albumin was withdrawn with a sterile injector and the eggs were left for incubation again. After incubation, a window (10 cm^2^) was created in the eggshell underlying the air sac, exposing the CAM in each egg. The windows in the shell were covered with laboratory film to prevent the embryos from drying out. Before treatments, the vascular regions of the eggs to be applied were taken at 1x magnification with a stereomicroscope (Leica) at 0 h. Embryonic eggs were then followed by negative control (1xPBS), L-EPS (750 and 1000 µg/mL) was treated with at least 3 parallel models with 50 µL of treatment on the CAMs^113^. Then, stereo microscope images were taken from the treated eggs at 1x magnification after 12 h and 24 h of incubation. CAM microscope images were evaluated for total vessel length with the length measurement tool in ImageJ Software (U. S. National Institutes of Health, USA).

### Statistics

Study results were statistically evaluated using SPSS 22.0 software and GraphPad Prism 8.0. Differences between groups were determined by T-test. In the scratch assay study, the differences between the average wound closure values ​​were determined by Multivariate Anova Dunnett. Differences between average production and expression levels in ELISA and qRT-PCR analyzes were determined by One-way Anova Post Hoc Tukey HSD. In the flow cytometry study, the differences between the mean cell division values ​​were determined with One-way Anova Dunnett. The difference between the average vessel length folds obtained in the CAM model was determined by Two-way Anova Bonferroni. Statistically significant was determined at the level of 95% (*p* < 0.05). All experiments were performed in triplicate, and mean values are presented. The results were given as mean ± standard deviation (SD).

## Data Availability

Data will be made available on request.The author from whom the data will be requested: demirrr.abdullah@gmail.com.
